# Immune Regulatory Activity of Vitamin D_3_ in Head and Neck Cancer

**DOI:** 10.3390/cancers5031072

**Published:** 2013-08-14

**Authors:** M. Rita I. Young, Terry A. Day

**Affiliations:** 1 Research Service, Ralph H. Johnson VA Medical Center, 109 Bee Street, Charleston, SC 29401, USA; 2 Department of Otolaryngology—Head and Neck Surgery, Medical University of South Carolina, 135 Rutledge Avenue, Charleston, SC 29425, USA; E-Mail: dayt@musc.edu; 3 Department of Medicine, Division of Hematology/Oncology, Medical University of South Carolina, 96 Jonathan Lucas Street, Charleston, SC 29425, USA

**Keywords:** head and neck cancer, HNSCC, immune modulation, immune suppression, suppressor cells, vitamin D

## Abstract

While vitamin D exhibits a multitude of cellular effects that can impact on cancer development and progression, this review focuses on its immune modulatory effects. These immune modulatory effects can be both direct and indirect. Compared to other cancer types, head and neck squamous cell carcinomas (HNSCC) have received less attention, but are a fascination immunologically because of the profound extent to which they inhibit immune defenses. This review describes the mechanisms of some of these immune inhibitory processes and how vitamin D can help overcome aspects of this immune suppression.

## 1. Induction of Immune Inhibitory Cells by HNSCC

Historically, treatment of head and neck squamous cell carcinoma (HNSCC) has been centered around varying combinations and sequences of chemotherapy, radiation therapy and surgery. FDA approval of chemotherapeutics has been limited. Although platinum-based agents were the standard of care for decades, docetaxel was the first chemotherapeutic approved by the FDA for use in the HNSCC population. However, chemotherapies often lead to short and long term toxicity. Cetuximab was the first biologic agent approved by the FDA for use in HNSCC. Despite the use of various therapies, including combinations of chemotherapy, surgery and radiation, survival rates have not changed dramatically over decades, while toxicity from treatment may be increasing. The 5-year survival of patients with HNSCC has remained at approximately 60%, although this percentage varies depending on the stage at which cancer was diagnosed [1]. Thus, new approaches remain necessary to improve treatment, reduce recurrence, prevent cancer and reduce side effects.

Immunotherapy in head and neck cancer can be used to modify and activate the immune system to prevent tumor progression, control tumor growth, and modify the host-tumor microenvironment. Immunotherapy is often aimed at reducing tumor recurrence, treatment of minimal disease and reducing toxicity. Unfortunately, HNSCC patients have profound immune defects that are associated with increased recurrence [2]. For example, lymph nodes of HNSCC patients are reduced in size and have diminished T-cell content [3]. T-cells from about one-third of HNSCC patients have been shown to be unresponsive to stimulation through the CD3/T-cell receptor [4]. HNSCC patients also have defects in maturation of dendritic cells, which are critical for stimulating tumor-specific T-cell reactivity, and approaches are being tested to stimulate dendritic cell differentiation and function [5,6,7]. The impact of immune depression in HNSCC patients on the clinical course of disease is indicated by the association between reduced T-cell function and poorer disease-specific survival [8]. The outcome for HNSCC patients with reduced levels of lymphocytes relative to levels of neutrophils is worse than for patients with an increased proportion of lymphocytes [9].

**Figure 1:**
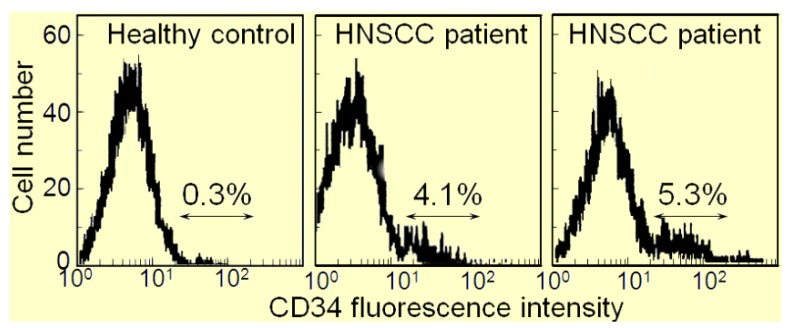
Increased levels of CD34^+^ cells in peripheral blood of HNSCC patients.

**Figure 2:**
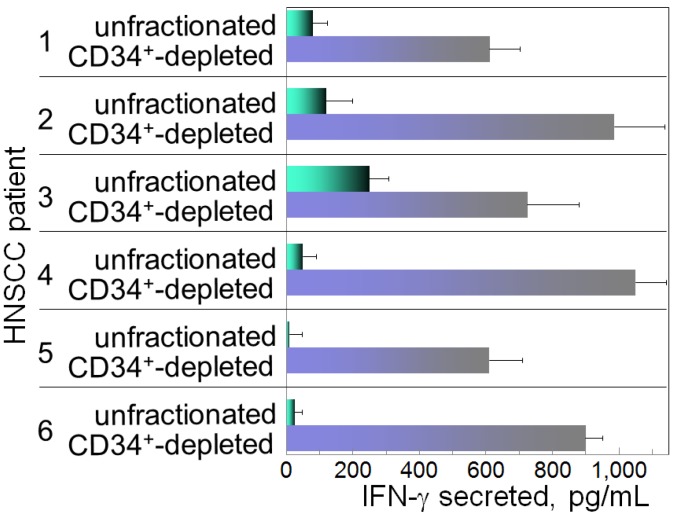
Inhibition of IFN-γ production in the presence of patients’ CD34^+^ cells.

The immune depression in HNSCC patients is caused not only by immune suppressive mediators produced by the HNSCC cells, but also the immune suppressive cells that they induce. Recent attention has focused on the contribution of M2 macrophages, Th2 skewed T-cells, Treg cells, myeloid-derived suppressor cells (MDSC) and CD34^+^ progenitor cells to HNSCC-induced immune dysfunction [10,11,12,13,14,15,16]. The CD34^+^ cells are hematopoietic progenitor cells that intensely express the CD34 marker, as opposed to the dimmer level of expression by endothelial cells. Their numbers are elevated in patients with HNSCC, and they exhibit non-specific suppression of T-cell function [17,18]. In healthy individuals, CD34^+^ cell levels are less than 1% of the peripheral blood mononuclear leukocyte population in contrast to patients with HNSCC, where they compose approximately 5% of the peripheral blood leukocyte population (Figure 1). Shown in Figure 1 are flow cytometric histograms demonstrating the low percentages of cells staining positive for CD34 in the peripheral blood of a representative healthy subject and the increased percentages of CD34^+^ cells in two separate HNSCC patients. The increased presence of these CD34^+^ cells is induced by tumor-derived granulocyte-macrophage colony-stimulating factor (GM-CSF) and they are chemoattracted into the HNSCC mass by tumor-derived vascular endothelial cell growth factor (VEGF) [19]. That these CD34^+^ cells are inhibitory to T-cell reactivity is demonstrated by the increased capacity of peripheral blood mononuclear cells to be activated to produce IFN-γ upon immunomagnetic depletion of CD34^+^ cells (Figure 2). Specifically, peripheral blood mononuclear cells from HNSCC patients were either unfractionated or were immunomagnetically depleted of CD34^+^ cells. Their activation to secrete IFN-γ in response to stimulation with anti-CD3 and low dose IL-2 was then measured by ELISA. Shown in Figure 2 for 6 different HNSCC patients are levels of IFN-γ produced by their mononuclear cells that were either unfractionated or from which CD34^+^ cells were depleted. In the presence of CD34^+^ cells, levels of IFN-γ that are released in response to stimulation through the T-cell receptor are lower than when CD34^+^ cells are depleted.

**Figure 3:**
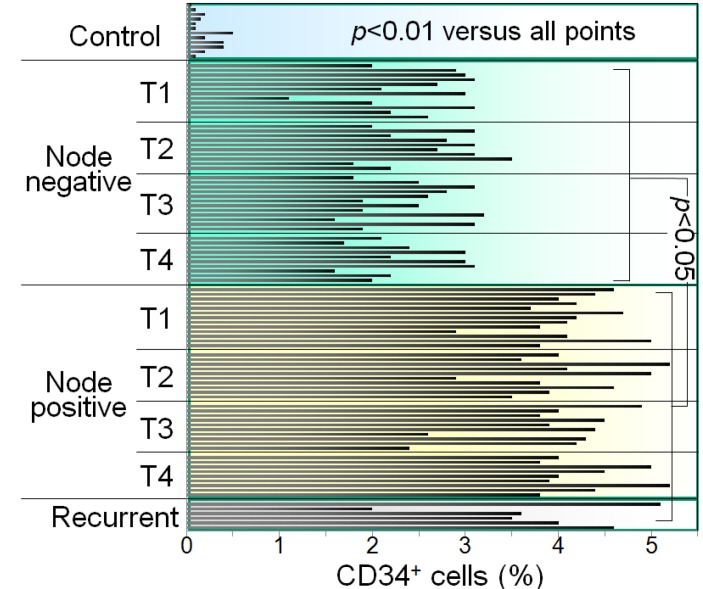
Levels of CD34^+^ cells are increased in the blood of HNSCC patients with nodal involvement compared to node-free patients.

The HNSCC patients were then evaluated for whether tumor stage was associated with the extent to which CD34^+^ cells were increased. When analyzed without regard to nodal involvement, patients with stage T1-T4 HNSCC have an increase in the percentage of CD34^+^ cells in the peripheral blood mononuclear cell population as compared to control patients, with no difference seen among groups with various stages of HNSCC [17]. What is most significant is the increase in the percentage of CD34^+^ cells in patients with node positive T1-T4 tumors as opposed to patients without nodal involvement (Figure 3). This was demonstrated by immunostaining peripheral blood mononuclear cells from HNSCC patients for CD34 and quantitating the proportion of CD34^+^ cells by flow cytometry. Shown are levels of CD34^+^ cells in the peripheral blood of patients, staged T1 to T4, either with nodal disease or that were node negative for HNSCC. Each bar represents results from an individual patient. Patients with node positive HNSCC have greater levels of CD34^+^ progenitor cells in their peripheral blood, regardless of the cancer stage. These increases in levels of CD34^+^ immune inhibitory cells could be due to increased mobilization from the bone marrow due to tumor production of GM-CSF. In fact, consistent with the increase in CD34^+^ cells in node positive HNSCC patients, they also have significantly higher levels of GM-CSF than those that are node negative [17]. The increase in CD34^+^ cells in HNSCC patients could also be contributed by defective maturation into more mature cell types. Consistent with this possibility are reports of HNSCC patients having defects in maturation of dendritic cells, which are critical for stimulating tumor-specific T-cell reactivity [5,20]. It is this latter observation that became the basis for developing a vitamin D-based treatment approach.

## 2. Overcoming Cancer-Induced Immune Inhibitory Mechanisms with Vitamin D

Vitamin D supplementation is becoming increasingly popular for its role in calcium metabolism, muscular function and prevention of autoimmune and cardiovascular diseases. Whether or not vitamin D metabolites impact on cancer development has been controversial and has been recently reviewed [21]. Studies have shown that the active metabolite 1α,25-dihydroxyvitamin D_3_ [1,25(OH)2D3] has clinical effectiveness in a hamster buccal pouch tumor model [22]. In mouse models, 1,25(OH)_2_D_3_ therapy can reduce the extent of metastatic disease and, when combined with adoptive immunity, reduces metastasis [23]. Contributing to the debate of the extent of anti-tumor effectiveness of vitamin D metabolites is the variability in the levels of vitamin D that are used in studies to assess its anti‑cancer potential. Nevertheless, studies have shown vitamin D supplementation improves survival of breast cancer patients and reduces the risk of developing breast or colorectal cancer [24,25,26]. Some studies have attributed the anti-cancer effects of vitamin D to inhibition of proliferation [24,25,26,27,28]. For example, mice fed a diet with only a low vitamin D content had increased prostatic epithelial cell proliferation, leaving them more prone to prostate cancer development [28]. Exposure of human endometrial epithelial cells to progesterone upregulated vitamin D receptor expression and, in turn, the expression of apoptosis-related proteins to result in cell cycle arrest [29].

While the mechanism by which 1,25(OH)_2_D_3_ exerts its anti-tumor effects has not been fully elucidated, it is clear is that vitamin D metabolites possess active immune regulatory properties, albeit these properties can be paradoxical. For example, 1,25(OH)_2_D_3_ suppresses the inflammatory effects of the Th1 responses in pulmonary tuberculosis, but simultaneously promotes macrophage bactericidal activity [30,31,32]. Vitamin D metabolites are protective against experimentally induced autoimmunity, and prevent dendritic, Tc1, and Th1 cell differentiation [30,33]. Vitamin D metabolites can exert anti-inflammatory effects to lessen radiation-induced lung inflammation, systemic lupus erythematosus and multiple sclerosis [34,35,36,37].

**Figure 4:**
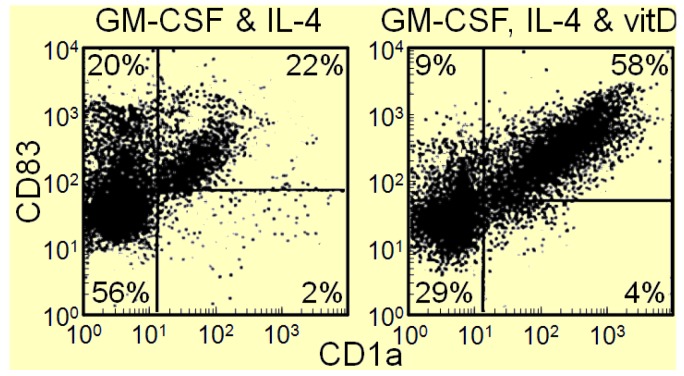
1,25(OH)_2_D_3_ increases differentiation of HNSCC patient blood-derived CD34^+^ cells into dendritic cells.

In contrast to the immune moderating effect of vitamin D metabolites in infectious and autoimmune disease setting, 1,25(OH)_2_D_3_ can activate the immune system in cancer patients and stimulate intratumoral immune infiltration [15]. These improvements in anti-tumoral activity in association with 1,25(OH)_2_D_3_ are intuitively in contrast to the Th2 promoting effects of 1,25(OH)_2_D_3_ in other settings. Since HNSCC patients have an accumulation of immune inhibitory CD34^+^ progenitor cells and a defect in dendritic cell differentiation [17,38], our studies aimed to determine if vitamin D metabolites could overcome the dendritic cell differentiation defect. The rational for determining if vitamin D metabolites would stimulated differentiation of immune inhibitory CD34^+^ progenitor cells into dendritic cells is based on prior demonstrations of vitamin D metabolites stimulating differentiation of other cell types [39,40]. In our studies, CD34^+^ cells were isolated from peripheral blood mononuclear cells of HNSCC patients and cultured with GM-CSF and IL-4 in the presence or absence of 1,25(OH)_2_D_3_. After 12 days of culture, cells were detached and immunostained for CD1a and CD83 to identify dendritic cells as those that stained positive for both markers. Results of these studies shown in Figure 4 indicate that adding the active metabolite 1,25(OH)_2_D_3_ to GM-CSF and IL-4 doubles the number of dendritic cells that differentiate from immune inhibitory CD34^+^ progenitor cells from the peripheral blood of HNSCC patients (Figure 4 data previously not published) [41,42]. A large proportion of the U.S. population is vitamin D deficient [43,44], which raises the question of whether vitamin D deficiency contributes to the defects in differentiation of immature CD34^+^ cells into mature dendritic cells in HNSCC patients.

**Figure 5:**
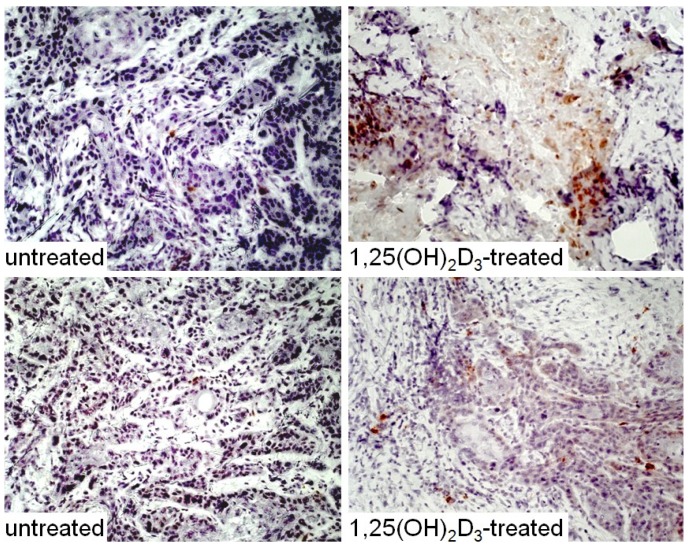
Increased levels of mature dendritic cells in HNSCC after treated with 1,25(OH)_2_D_3_.

**Figure 6:**
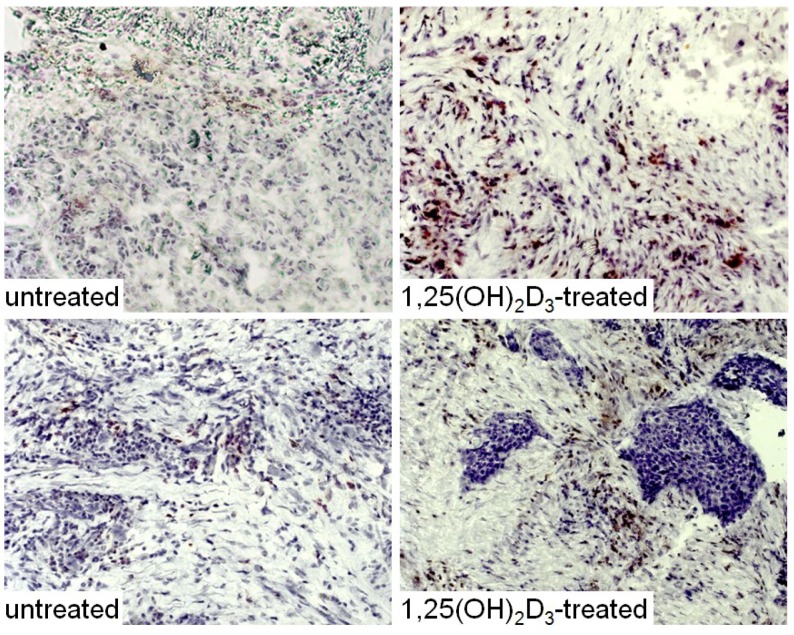
Increased levels of CD4^+^ cells in HNSCC of patients treated with 1,25(OH)_2_D_3_.

**Figure 7:**
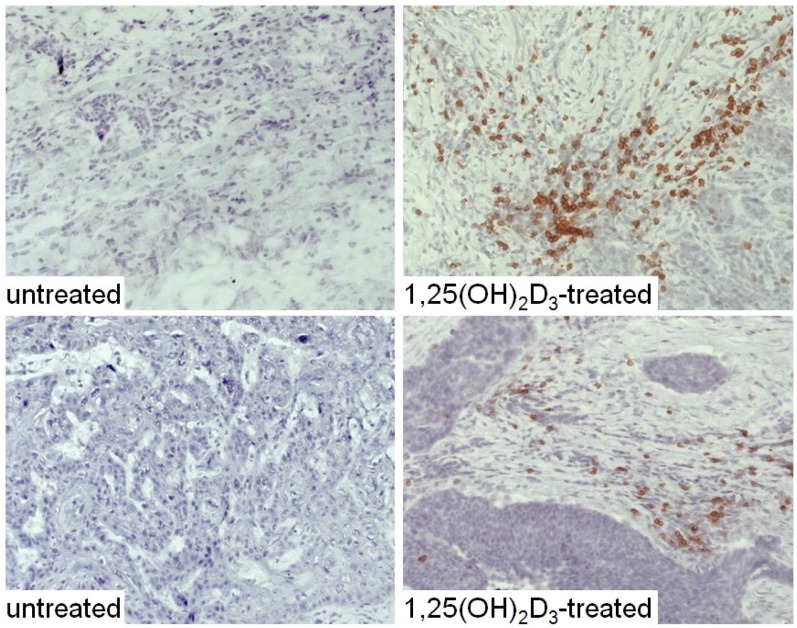
Increased levels of CD8^+^ cells in HNSCC of patients treated with 1,25(OH)_2_D_3_.

**Figure 8:**
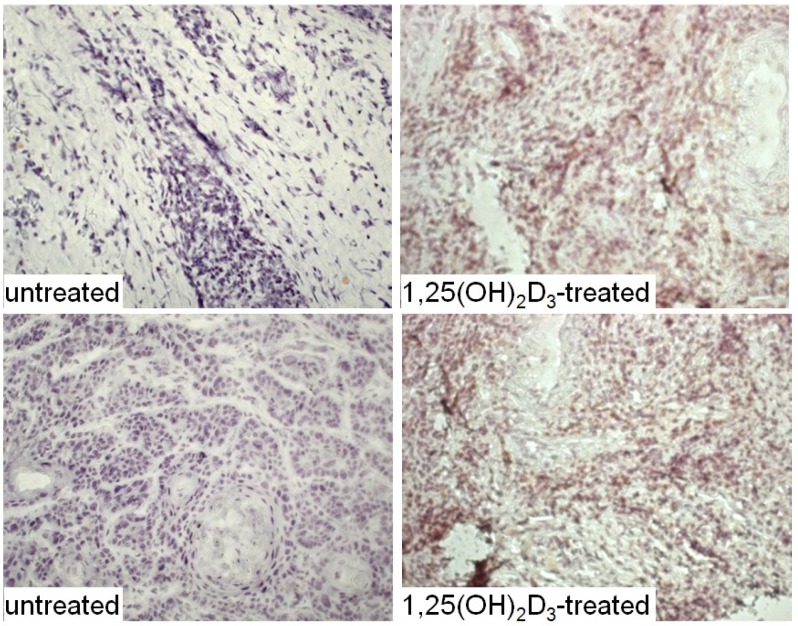
Increased levels of cells expressing the activation marker, CD69, within HNSCC tissue of patients treated with 1,25(OH)_2_D_3_.

Our *in vitro* studies showing enhancement of dendritic cell differentiation from CD34^+^ cells of HNSCC patients supported determining whether treatment of HNSCC patients with 1,25(OH)_2_D_3_ would reduce levels of CD34^+^ immune suppressive cells, increase the levels of mature dendritic cells and increase levels of stimulated T-cells. For these studies, patients with newly diagnosed HNSCC received treatment orally for 3 weeks with 4 μg 1,25(OH)_2_D_3_ for each of 3 sequential days and then receive no treatment for 4 days. This was repeated for 3 weeks, the average duration between diagnosis and surgical treatment. HNSCC specimens that were excised as part of the patients’ surgical treatment were collected for immunohistochemical analyses from patients after the 3-week period of 1,25(OH)_2_D_3_ treatment (Figure 5, Figure 6, Figure 7 and Figure 8, data not previously published). Surgically excised HNSCC specimens from untreated patients were used as controls. This trial with HNSCC patients showed that 1,25(OH)_2_D_3_ diminishes peripheral blood and intratumoral levels of immunosuppressive CD34^+^ cells and increases levels of mature dendritic cells, as identified by positive staining for CD‑LAMP (Figure 5) [15,45]. Shown in Figure 5 are representative immunostained tissues from two separate untreated patients and two patients that received 1,25(OH)_2_D_3_ prior to surgery.

**Figure 9:**
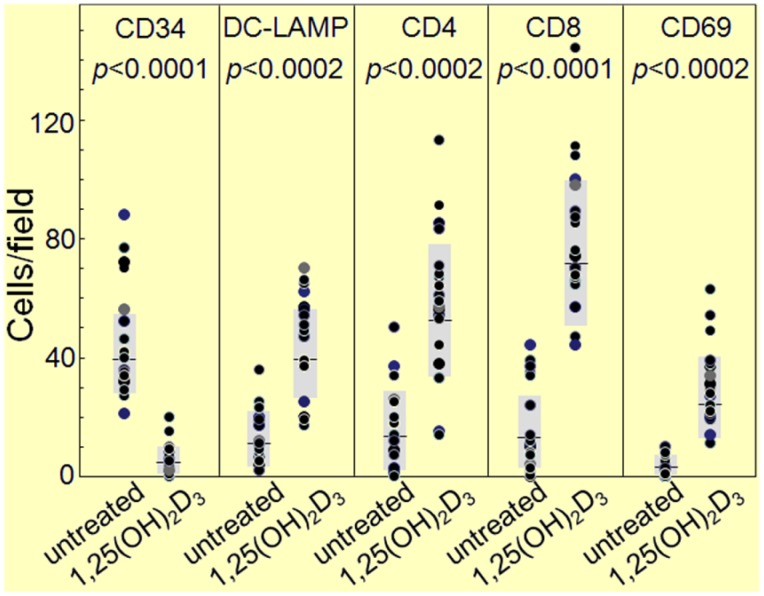
Quantitation of increased immune infiltration within HNSCC tissue of patients treated with 1,25(OH)_2_D_3_.

The HNSCC tissue from patients that were either untreated or received 1,25(OH)_2_D_3_ treatment between diagnosis and surgical treatment were also immunostained for levels of immune infiltrating cells. Coinciding with the decline in CD34^+^ cells and the increased levels of mature dendritic cells was an increase in CD4^+^ and CD8^+^ cells within the tumor tissue, and an increase in intratumoral levels of T-cells expressing the activation marker, CD69 (Figure 6, Figure 7 and Figure 8; summary in Figure 9). Figure 6, Figure 7 and Figure 8 show representative immunostained tissues from two separate untreated patients and two patients that received 1,25(OH)_2_D_3_ prior to surgery. Figure 9 shows the quantitative increase of these immune cells within the HNSCC tissue following 1,25(OH)_2_D_3_ treatment.

Clinical outcome of the 1,25(OH)_2_D_3_ treatment was also monitored by the time to cancer recurrence. Totally unexpected and most surprising was that the time to cancer recurrence following surgical treatment was increased by over 3-fold in the group receiving 1,25(OH)_2_D_3_ as opposed to the group of untreated patients [45].

## 3. Interplay of Vitamin D and COX-2 in Immune Modulation in Cancer

The studies described immediately above associate the anti-cancer effects of 1,25(OH)_2_D_3_ with its capacity to overcome the defect in maturation of progenitor cells toward dendritic cells to, in turn, stimulate increased intratumoral T-cell influx. However, there are other immunological means by which vitamin D could enhance immune reactivity. Studies have shown vitamin D can diminish levels of COX-2 [46,47,48]. For example, 1,25(OH)_2_D_3_ can normalize overexpressed levels of COX-2 in estrogen-deficient rats [49]. Overexpression of COX-2 by both HNSCC as well as several immune inhibitory cell types that they induce contributes to the immune dysfunction in HNSCC patients [50,51,52,53]. These include tumor-associated macrophages [54] and endothelial cells, which we recently identified to be induced by HNSCC [51]. Studies have shown immune restoration and prolonged survival associated with COX-2 inhibition [55,56]. In contrast to the inhibitory effect of vitamin D metabolites on COX-2, a low calcemic 1,25(OH)_2_D_3_ analog, EB1089, has been shown to upregulate COX-2 gene expression in a human HNSCC cell line [57]. Consequently, because of the immune inhibitory role of COX-2 expression and the inter-relationship between COX-2 and vitamin D, the immune regulatory role of vitamin D cannot be fully separated from its modulation of PGE_2_.

## 4. Additional Immune Modulatory Mechanisms of Vitamin D That Can Impact on Cancer Development and Progression

In addition to modulating COX-2 activity, vitamin D can also regulate other inflammatory processes. There has been extensive discussion about the pro- and anti-cancer effects of inflammation. Levels of the pro-inflammatory Th17 population of cells are increased in patients with HNSCC as well as in animal models, especially those with premalignant oral lesions [58,59]. Studies with several tumor models have associated Th17 cells with having an anti-tumor effect, although they can also be pro-angiogenic [59,60]. There have also been indications that IL-17-producing inflammatory Th17 cells can contribute to tumor-induced immune inhibition and enhanced tumor development. For example, Th17 cells can differentiate into immune inhibitory Treg cells in cancer patients [61]. Furthermore, Th17 cells can facilitate cancer development [62]. While studies on the inter-relationship between Th17 cells, vitamin D and progression of HNSCC have not been done, there have been studies related to other disease states such as in animal models for multiple sclerosis demonstrating vitamin D treatment reduces levels of IL-17 and, in turn, reduced the contribution of Th17 cells to disease progression [35]. This coincides with studies showing that multiple sclerosis patients in relapse have lower levels of vitamin D as compared to patients in remission. Furthermore, 1,25(OH)_2_D_3_ can reduce levels of the pro-inflammatory cytokines, IL-6 and IL-17 [34]. In separate studies, the addition of 1,25(OH)_2_D_3_ to a mixture of peripheral blood mononuclear cells and tumor cells reduced levels of the inflammatory cytokines TNF-α and IL-6 and, to a lesser extent, the immune inhibitory cytokines, IL-10 [63]. Thus, if inflammation is pro-tumorigenic, the anti-inflammatory effects of vitamin D could be contributing to its anti-tumor effects.

## 5. Conclusions

To advance the field of how vitamin D might impact on cancer development and progression, gene expression analyses are now examining molecular pathways that might contribute to the understanding of how vitamin D may be linked to reduced cancer. Such studies have shown that a large proportion of gene sets correlating with plasma vitamin D levels are associated with immune function [64]. However, these studies have also shown the diversity of pathways, including those that modulate tumor cell growth and apoptosis are also regulated by vitamin D [57,65]. Thus, the components of the spider web of means by which vitamin D may exert inhibitory effects on cancers cannot be separated when deciphering mechanisms underlying its *in vivo* anti-cancer activity. Key is the interplay among the multitude of pathways that are modulated by vitamin D. Among these are the immune regulatory activities of vitamin D that can impact on HNSCC cancer development and progression.
